# Correction: Mutations in LRRK2 linked to Parkinson disease sequester Rab8a to damaged lysosomes and regulate transferrin-mediated iron uptake in microglia

**DOI:** 10.1371/journal.pbio.3001621

**Published:** 2022-05-04

**Authors:** Adamantios Mamais, Jillian H. Kluss, Luis Bonet-Ponce, Natalie Landeck, Rebekah G. Langston, Nathan Smith, Alexandra Beilina, Alice Kaganovich, Manik C. Ghosh, Laura Pellegrini, Ravindran Kumaran, Ioannis Papazoglou, George R. Heaton, Kirsten Harvey, Rina Bandopadhyay, Nunziata Maio, Changyoun Kim, Matthew J. LaVoie, David C. Gershlick, Mark R. Cookson

Dr. Kirsten Harvey should be included in the author byline. She should be listed as the fourteenth author, and her affiliation is: Department of Pharmacology, UCL School of Pharmacy, University College London, London, UK. Contributions of this author are as follows: Funding Acquisition and Supervision.

The Funding Disclosure should contain Dr. Kirsten Harvey’s funding details. Please see the correct Funding Disclosure below:

This work was supported in part by the National Institutes of Health, NIA, Intramural Research Program (MRC), by NIH extramural grant NS110188 (MJL), the Wellcome Trust grant 106805/Z/15/Z (GRH, KH, MRC), the Medical Research Council grant MR/M00676X/1 (KH) and the NIA IRP Postdoctoral grant scheme (AM). The funders had no role in study design, data collection and analysis, decision to publish, or preparation of the manuscript.
