# Supplementary material for: Mutations in LRRK2 linked to Parkinson disease sequester Rab8a to damaged lysosomes and regulate transferrin-mediated iron uptake in microglia
Source: PLoS Biol. 2021 Dec 16;19(12):e3001480. doi: 10.1371/journal.pbio.3001480 (PMC8675653; doi:10.1371/journal.pbio.3001480)
Supplement: Attachment — Submitted filename: Response to Reviewers.docx [file pbio.3001480.s014.docx]

REVIEWS:

Reviewer #1: In this manuscript, the authors conclude that mutant LRRK2 sequesters Rab8a in lysosomes and dysregulates TfR trafficking, leading to iron accumulation in brain striatal microglia of G2019S LRRK2 mice. This is an important area of broad interest to the readers of PLoS, but as discussed in detail below, the present story would require significant additional work before it could be considered for presentation in PLoS Biology; the conclusions are premature.

1. First the authors conclude that pathogenic LRRK2 causes Rab8a to be localized in lysosomes. Hilfiker has shown that Rab8a vesicles are concentrated near the centriole and lysosomes may also accumulate at the same location as independent structures. Proof of co-localization would require addition of a microtubule depolymerizing drug such as nocodazole to be sure that separate structures were not simply near each other. What fraction of total Rab8a co-localizes with a lysosomal marker ± nocodazole?

We thank the reviewer for this suggestion. We have now performed a detailed analysis of centrosomal vs lysosomal Rab8a localization in the context of LRRK2 mutations as well as nocodazole treatment, and have included the new data in the revised manuscript. We found that, much like observed by Hilfiker and colleagues, mutant LRRK2 induces translocation of Rab8a to the perinuclear region. In our experiments, Rab8a is recruited by mutant LRRK2 to lysosomes proximal to the centrosome (Fig 1E). We performed analysis of Rab8a presence near the centrosome as performed by Hilfiker and colleagues (% of Rab8a staining in a 2.2µm diameter circle ROI enclosing centrosomal staining versus whole cell Rab8a staining) and report enhanced localization of Rab8a proximal to centrosomes with mutations that is rescued by nocodazole treatment (Fig 1E, F). Furthermore, as suggested by the reviewer, we analyzed the fraction of Rab8a that localizes with Lamp2 following nocodazole treatment, by assaying the Manders overlap coefficient. We report that around 50% of total Rab8a (Manders coefficient 0.5) localizes with Lamp2 in the presence of G2019S LRRK2 and this is decreased to ~30% with nocodazole (Fig 1G, H). These data suggest that Rab8a associates with LRRK2-positive perinuclear lysosomes proximal to the centrosome and microtubule integrity can influence this association, presumably by impairing anterograde transport and lysosomal sequestration. We have detailed these data in the results and discussion sections of the manuscript.

2. The authors use a metric, "cells with sequestered Rab8a vesicles", monitoring the number of Rab8a vesicles per cell. At this level of resolution they are not really measuring vesicle numbers as the vesicles are below the resolution limit of this type of microscopy. Also of concern is the fact that when two adjacent cells are shown, they do not show the same phenotype as the cells being outlined. Nevertheless, a more appropriate metric would be percent of cell area occupied by labeled pixels in the various conditions to indicate possible concentration.

We thank the reviewer for the opportunity to clarify this in our manuscript. We agree with the reviewer that the term “sequestered Rab8a vesicles” is not appropriate for this type of analysis and have amended this. Originally, the high-content imaging analysis of Rab8a sequestration was done on the cellomics platform where we used the “spot detection” algorithm to detect Rab8a foci and quantify the percentage of cells that exhibit Rab8a structures larger than ~4µm^2. This has now been outlined in the text (results and methods sections). We have now repeated these experiments and analyzed the colocalization of Rab8a with LRRK2 and Lamp2 by confocal microscopy (ZEISS 880, 63x) and have generated Manders overlap coefficient measurements (Fig 1B and C). Thus, the high-content imaging analysis was moved to supplementary fig 2B, where we outline the effect of a series of PD-linked mutations on Rab8a sequestration.

3. In E, the authors detect a Rab8a decorated lysosome—and in D use arbitrary units to document co-localization between Rab8a and LRRK2. What percent of pixels stained for each marker co-localize with the other marker and vice versa? "High throughput colocalization analysis" doesn't match the data shown so the authors need to be more hands on in their analysis and cells need to be viewed and analyzed at high magnification (100X lens), unclustered. A single Airyscan image shows Rab8a lysosome decoration but in their prior work, they reported that in the absence of LLOME, this was only seen for a very few percent of total lysosomes (ref 17).

Our original colocalization analysis was done by high-content imaging on the cellomics platform using a 10x objective, and this had been presented in panels C and D of the original submission. As outlined in our response to point 2 above, following the suggestion by the reviewer we have now performed Manders overlap coefficient analysis between Rab8a, Lamp2 and LRRK2 on confocal images obtained on a ZEISS LSM 880 using a 63x oil objective. These analyses are now presented in fig 1B and C. We observe a significant increase in Rab8a colocalization with LRRK2 and Lamp2 in cells expressing G2019S or R1441C LRRK2 compared to WT LRRK2. As suggested by the reviewer, we quantified the percentage of cells that exhibit Rab8a sequestration to lysosomes and observed that about 50% of cells expressing R1441C LRRK2 and 30% of cells expressing G2019S LRRK2 show Rab8a lysosomal association (Fig S2 C). This is in accordance with our prior published research where we reported that about half of primary astrocytes transiently expressing LRRK2 show LRRK2-positive lysosomes.

4. Figure 2. Here, the authors report what they have already published—Rab8a on LLOME lysosomes. Line scans are used to document lack of co-localization of cathepsin D with LAMP2 in LRRK2 expressing cells. A line scan is a poor mode of reporting co-localization as all data not under the line is lost. Here, again, it is essential to ask what fraction of total label co-localizes.

We have now performed Manders overlap coefficient analysis to assay the fraction of LRRK2 positive lysosomes that contain Cathepsin D. While there is a significant increase in the Manders overlap coefficient between mutant LRRK2 and Lamp2 staining reflecting association with lysosomes, LRRK2 and Cathepsin do not exhibit significant colocalization suggesting that these are lysosomes with low degradative capacity (Fig 2B).

5. Next the authors confirm that pRab8a cannot bind Rabin8 but binds MICAL-L1. This confirms a report by Steger., 2017 using mass spec IP, and is consistent with the colocalization in cells shown here ±LRRK2.

We thank the reviewer for this note. While we had cited the Steger paper elsewhere in the text, we now also cited it in this section, outlining that our data are consistent with this published work.

6. Figure 4 shows an apparent coalescence or local concentration of TfR containing membranes. These are not necessarily lysosomes, they are simply concentrated in one cell region and would require a nocodazole experiment to determine if they are actually the same membrane compartment as lysosomes. Again, because the vesicles are not resolved, area occupied by TfR might be more revealing?

To test whether the coalescent TfR staining that we observe with mutant LRRK2 associates with lysosomes we repeated these experiments and analyzed the fraction of TfR colocalizing with the late endosome/lysosome marker Lamp2 (Fig S4). We report a significant increase in colocalization between TfR and Lamp2 in cells expressing R1441C (~50% of whole cell TfR staining) and G2019S LRRK2 (~40%) compared to WT LRRK2 expressing cells (Fig S4). Our data are consistent with published research that reports perinuclear localization of TfR in cells expressing kinase hyperactive mutant LRRK2 (Dhekne et al, 2018; eLife). and this has now been cited in the text. To further examine the observation that TfR is recruited by LRRK2 to damaged lysosomes, we treated cells with the lysosomal destabilizing agent LLOMe and analyzed intracellular localization by Airyscan (Fig S4). We observe TfR recruitment in LRRK2+ and Rab8a+ membranes consistent with damaged lysosomes that we presented in previous work (Bonet-Ponce et al, 2020). Following the suggestion of the reviewer, we examined TfR localization under nocodazole treatment. Depolymerizing the microtubule network produced a dispersed lysosomal phenotype and reversed the perinuclear sequestration of TfR and colocalization with Lamp2 (Fig S4). This is consistent with previous studies that report the disruption of TfR transport near the nucleus in cells treated with nocodazole (Hoepfner et al, 2005; Sakai et al, 1991). Our data suggests that TfR is sequestered near the cytocenter and associates with lysosomes is dependent on microtubules.

7. In 4E, the authors imply that cells take up more transferrin over time. This could be because they are slower at recycling the transferrin back into the medium or they take it up for a longer period of time. Experiments need to be included that monitor recycling (chase out the label and monitor decrease in fluorescence) and also quantify TfR surface numbers at steady state to provide a meaningful answer related to TfR function upon LRRK2 expression. In addition, the effect of a LRRK2 inhibitor on a single mutant cell type would need to be included for the results to be meaningful and know that differences were due to LRRK2.

We thank the reviewer for this suggestion and the opportunity to enhance our work. We performed transferrin uptake and recycling assays, utilizing Alexa Fluor 568 conjugated transferrin, in both HEK293 cells transiently expressing WT and mutant LRRK2, as well as iPSC-derived microglia, to address recycling dysregulation. In HEK293T cells we detected a modest increase in internalized transferrin levels in the first 15 minutes of uptake by G2019S LRRK2 (Fig 4F) which was rescued by MLi-2 treatment to levels comparable to WT LRRK2 expressing cells. In iPSC microglia we detected a trend of an increase in Tf levels but this was not significant in this model (Fig S6). The Tf recycling assays revealed higher initial transferrin levels at T0 in G2019S LRRK2 expressing HEK293 cells compared to WT, while this difference was not evident at later time-points (Fig 4G). In iPSC microglia we detected a subtle but significant increase in Tf levels and slower clearing in G2019S LRRK2 cells in the presence of LPS compared to WT cells (Fig S6). We also examined the levels of surface-bound transferrin by high-content analysis of fixed but not permeabilized HEK293 cells stained for TfR, and detected no significant difference between G2019S LRRK2 and WT LRRK2 expressing cells (data not shown). These data, cumulatively suggest subtle dysregulation of transferrin recycling driven by LRRK2 mutations. The fact that only around a third of cells expressing G2019S LRRK2 show Rab8a recruitment to lysosomes may account for the lack of difference detected in surface TfR. We have added these results in the text and expanded the discussion section.

8. Next the authors detect an increase in iron accumulation. If TfR mediated, this could only happen if there was more recycling to increase the "bucket brigade" of TfRs bringing in iron; this would be opposite of what one would predict from Rab phosphorylation? Again this is why the authors need to better characterize the entire transferrin cycle.

As outlined in point 7 above, we detect TfR accumulation near the nucleus and a subtle dysregulation of transferrin recycling in HEK293 and iPSC microglia carrying LRRK2 mutations. We agree with the reviewer that our data do not demonstrate how TfR trafficking dysregulation leads to iron accumulation. It is important to note that in the ICP-MS experiment we extracted and assayed whole-cell iron levels irrespective of which iron pool may be driving the observed upregulation in G2019S LRRK2 expressing cells. Lysosomes are physiological stores of Fe(II) and lysosomal impairment or dysregulation of their ability to acidify leads to perturbations in iron homeostasis. Based on out data, we believe that while the net effect of LRRK2 on TfR recycling may be modest, as reflected by our newly added experiments, LRRK2 mutations may be dysregulating the dynamics of TfR transport to lysosomes and the integrity of the target lysosomal membranes, thus affecting lysosomal iron stores. A section has been added in the discussion to expand on our hypothesis and outline the different scenarios that could lead to iron upregulation, along with hypotheses that remain to be tested. This is of high interest to us and we are now pursuing experiments to understand the role of LRRK2 on the dynamics of iron transport, delivery to mitochondria by endosomes through kiss-and-run events, and storage in lysosomes.

9. In Fig. 5 the authors used their previous RNA seq data and found lots of endolysosome hits correlating with inflammation but this is not a surprise and has been reported broadly elsewhere. They injected wild type or LRRK2 KO mice with LPS and looked at microglia; KO animals had fewer microglia but that could be due to independent pathways and does not reveal much. Next they looked at transferrin containing compartments in microglia ± mutant LRRK2 ± LPS; the structures and overlap is not meaningful without nocodazole to resolve the overlapping labeling, and overlap at steady state may not be indicative of trafficking differences.

We thank the reviewer for these suggestions. We have now included data to assay TfR trafficking differences in iPSC-derived microglia carrying G2019S LRRK2. To this end, we performed Tf uptake and recycling assays in conditions of -/+ LPS treatment, and assayed Tf levels by high-content imaging, as outlined in point 7 above. We detected a subtle upregulation in the levels of transferrin in washout conditions suggesting impairment in recycling (Fig S6). This, together with the observed increase in the TfR colocalization with lysosomal and proximity to the nucleus in G2019S cells under LPS treatment, support a model of dysregulated TfR trafficking driven by LRRK2 mutations.

10. Finally, the authors saw significant differences in iron accumulation in G2019S brains—this may be far downstream of TfR due to an indirect lysosomal defect.

We agree with the reviewer and have commented on this in the discussion, expanding on the possible pathways that may be driving such a divergence in iron deposition while outlining the limitations of our observations in terms of a TfR-dependent phenotype.

Reviewer #2: The manuscript by Mamais and colleagues describes effects of pathogenic LRRK2 on transferrin receptor recycling and iron accumulation in microglia. Whilst generally an interesting manuscript which warrants publication in PLoS Biology, I have several comments which need addressing as outlined below:

Major:

1. The authors validate the Rab8a-specific antibody (Figure S1) which they subsequently use to show accumulation of Rab8 on dysfunctional lysosomes in the presence of pathogenic LRRK2 expression. For validation purposes, they show an absence of Rab8a signal upon RNAi of Rab8a by ICC with the D22D8 antibody, and a knockdown of Rab8a protein as assessed by Western blotting. Please also show successful knockdown of Rab8a by Western blotting for the other two antibodies (ab128022 and ab188574) which do not show a decrease in staining upon RNAi of Rab8a (Figure S1).

We thank the reviewer for this comment. We have now included WB data of Rab8a siRNA treated cells probed with the D22D8 and the ab188574 antibody, while we removed the ICC of ab128022 since it is not commercially available anymore. The ab188574 showed a clear band at ~25kDa as predicted but it also detected higher MW non-specific bands that were also present following Rab8a knockdown, which may explain why there is some background staining in ICC (Fig S1).

As indicated in materials and methods, ICC was performed in the absence of detergent. Given that the ab188574 antibody has been reported to be specific for Rab8a as assessed by Western blotting using Rab8a KO cell lysates, the authors should also evaluate whether the three antibodies shown in Figure S1 display specific staining in the presence of detergent.

We thank that reviewer for bringing this to our attention. We had incorrectly omitted the permeabilization step in our methods section. We confirm that all ICC was done with a 10 min in 0.2% Triton X-100/PBS permeabilization step between fixation and blocking, and this has now been updated in the methods section.

2. The authors show that pathogenic LRRK2 expression causes lysosomal damage, associated with the accumulation of Rab8a and LRRK2 on dysfunctional, enlarged lysosomes (Figure 1). These findings are in contrast to other studies suggesting that chemical induction of lysosomal damage is required to trigger accumulation of LRRK2 and Rabs on enlarged, dysfunctional lysosomes (Eguchi et al., PNAS, 2018). The authors should discuss their findings in the context of other published data. In addition, they show reversal of Rab8a/LAMP2 colocalization and Rab8a/LRRK2 colocalization by application of LRRK2 kinase inhibitor MLi2 for 1 h. Is the observed lysosomal enlargement also reverted?

We thank the reviewer for this comment. As noted by the reviewer, other studies, including previous work from our group, have shown that LRRK2 is recruited to damaged lysosomes along with Rab GTPases (Bonet-Ponce, 2020). While this is enhanced by LRRK2 mutations and lysosomotropic agents, it is in fact also observed at a low rate with WT LRRK2 under normal conditions (Bonet-Ponce, 2020). While in our present work we have not directly assayed whether lysosomal size is affected by LRRK2 mutations, other studies have reported enlarged and damaged lysosomes in the context of LRRK2 mutations in different cell models (Hockey et al, 2015; j Cell Sci; Henry et al, 2015, HMG). Thus, we believe our work is consistent with published research and adds valuable information on the effects of lysosomal LRRK2 association in the context of LRRK2 mutations.

3. The authors show (Figure 3) colocalization of Rab8 with MICALL1 in cells expressing I2020T mutant LRRK2, but not wt LRRK2. The data presented here are not convincing, since Rab8a and MICALL1 also seem to colocalize in wildtype LRRK2 expressing cells. The authors should perform similar experiments as shown in Figure 1, determining colocalization of Rab8a with MICALL1, MICALL1 with LRRK2, and MICALL1 with LAMP2. The latter will confirm whether MICALL1 indeed is recruited to damaged lysosomes in the presence of pathogenic, but not wildtype LRRK2 expression.

We thank the reviewer for this comment. In fig 3 we explored the effect of LRRK2 mutations on MICAL-L1 as this is an established interacting partner of Rab8a, localizing in recycling tubular endosomes with Rab8a and regulating the endocytic recycling compartment (Sharma et al, 2009; Mol Biol; Kobayashi et al; 2014 Biol Open). We have now added confocal analysis of MICAL-L1 localization and detailed colocalization analysis between MICAL-L1, LRRK2 and Lamp2 in cells expressing WT or mutant LRRK2 (Fig S3). We report that endogenous MICAL-L1 shows minimal colocalization with WT LRRK2 but this is significantly enhanced with R1441C and G2019S LRRK2 that recruit MICAL-L1 in a fashion similar to Rab8a. Furthermore, colocalization of MICAL-L1 with Lamp2 is also significantly increased in mutant LRRK2 expressing cells supporting lysosomal association.

4. The authors describe clustering of TfR-positive vesicles in cells overexpressing pathogenic LRRK2 (Figure 4). Please show whether this is reverted by application of LRRK2 kinase inhibitor MLi2. Importantly, they show that these structural changes are associated with increased sequestration of fluorescently-labelled internalized transferrin. Is this reverted by MLi2?

We thank the reviewer for this suggestion and the opportunity to enhance our study. We have now performed fluorescent transferrin uptake assays and have analyzed Tf distribution at T10 of uptake, in cells transiently expressing WT or G2019S LRRK2 -/+ MLi-2 (Fig S4 D). Our results suggest Tf clustering in G2019S LRRK2 expressing cells compared to WT LRRK2 cells, as revealed by a decrease in the mean distance between Tf vesicles (Fig S4). Furthermore, this was rescued by treatment with MLi-2 prior to Tf loading supporting a LRRK2 kinase activity mediated effect. These new data have now been added to the manuscript.

5. Whilst expression of all pathogenic LRRK2 mutants correlates with clustering of TfR-positive vesicles and an increase in internalized transferrin levels over time, the ICP-MS determination (Figure 4I) only reveals increased iron levels in cells expressing G2019S LRRK2. How do the authors explain this discrepancy?

We thank the reviewer for raising this point. We think that different transfection efficiency and LRRK2 expression levels may be driving a discrepancy between the LRRK2 mutant expressing cells in some biochemical assays. In ICP-MS, whole cell pellets were processed and analyzed, and we have previously observed that the R1441C and Y1699C LRRK2 constructs show lower LRRK2 protein expression levels compared to G2019S LRRK2 in HEk293 cells (Mamais et al, 2014). To tackle this issue we are now optimizing live iron imaging techniques by utilizing commercially available live imaging dyes that can be used for intracellular Fe2+ detection. This approach can be used in a high content imaging setup where we can monitor LRRK2 expression and localization in parallel.

6. Figure 5: the authors show RNA-seq data of wildtype primary microglia treated with either LPS of preformed a-syn fibrils in culture, and mention that the shared hits betweeen the two treatments reveal enrichment for endosomal and lysosomal pathways. Figure 5C shows the top hits related to endosomal (not lysosomal) pathways, so this statement in the result section is confusing. Similarly, they show single cell RNA-seq data from isolated microglia of wildtype versus LRRK2 KO mice upon either PBS or LPS injection. They show differences between resting and activated microglia in both cases, and related to various processes (Figure 5H). Are the genes depicted in Figure 5I the top hits (eg FTH1), or how do these genes relate to the gene ontology analysis of Figure 5H?

The heatmap in fig 5I was constructed on a curated list of genes that are significantly upregulated by inflammation, and are involved in endolysosomal processes and iron homeostasis. It represents the differential expression of a subset of top hits with inflammation. We have now elaborated on this in the text.

7. The authors use iPS-derived microglia from control and G2019S LRRK2 carriers to show clustering of TfR-positive vesicles which display increased size, but the relevance of these observations for TfR-mediated iron uptake and accumulation is not shown. Importantly, the authors should determine whether the structural observations correlate with altered internalization of fluorescently-labelled transferrin in these cells as well, similar to what they observed when overexpressing LRRK2 in HEK cells (Figure 4). Also, does LPS treatment of iPS-derived microglia in vitro alter protein levels of TfR or FTH1?

We thank the reviewer for this suggestion. We have now performed Tf uptake and recycling assays on iPSC-derived microglia to test whether LRRK2 G2019S affects transferrin trafficking. We found that the rate of uptake was comparable between WT and G2019S LRRK2 cells and did not detect accumulated Tf levels as in HEK293 cells. In recycling assays the G2019S LRRK2 cells showed slower Tf clearance albeit the effect was subtle (Fig S6). In the time we had to review our work, and due to covid-related lab restrictions, we were not able to run WB analysis of these cells for TfR and FTH1 levels as the yield of differentiation is limited and we gave priority to high-content Tf imaging. Based on mRNA analysis of isolated microglia as well as in vivo data presented in figure 8, we expect FTH1 upregulation following LPS treatment.

8. The authors show increased iron deposition in microglia in G2019S knockin mice as compared to controls upon intrastriatal LPS injection, associated with increased levels of FTH1 and Tf (Figure 7 and Figure 8). What about differences in iron levels in the absence of LPS injection, especially in the SN?

We did not show these images because there was very little iron staining to be imaged, both in not injected animals as well as in PBS injected controls used in figure 7. We believe that this is to be expected as iron levels are tightly regulated and in physiological conditions we would expect minimal iron deposition.

Their data indicate that LPS treatment causes increased iron deposition in activated microglia in G2019S mice as compared to control. This may reflect altered iron handling in microglia in G2019S versus control mice (perhaps due to differences in microglial activation), but how this relates to the described increases in iron in dopaminergic neurons, as relevant for PD pathogenesis, remains unclear. In this context, the discussion would benefit from restructuring as well, especially with respect to the relevance of iron deposition in PD brains, as the authors do not provide evidence of altered iron handling in SN, in the absence of LPS, in the G2019S knockin mice as compared to control mice.

We have now restructured the discussion and outlined the limitations of our findings in terms of relevance to iron deposition in PD.

Minor:

1. please mention time and concentration of LLOME employed.

We did 1mM LLOME for 4hrs. This has now been amended in the methods section, thank you.

2. Results: the authors mention "Given the above data showing that LRRK2 phosphorylation prevents Rab8a activation and redirects Rab8a and MICAL-L1 away from the ERC,...". What evidence do the authors have that phosphorylation prevents Rab8a activation? If based on differential interactions of Rab8a mutants, this statement should be deleted, since these mutants have been shown not to mimic the phospho-status of the Rab protein.

We thank the reviewer for this comment. We agree with the reviewer that our understanding of LRRK2 mutations on Rab8a activation is limited and have now deleted this sentence.

Reviewer #3: Overall: The authors present an elegant set of studies demonstrating that a) LRRK2 GOF mutations lead to kinase-dependent sequestration of Rab8a in lysosomes, b) GOF mutations dysregulate iron transport by driving the iron-binding transporter transferring into Rab8b-containing lysosomes. The authors go on to show that microglia derived from iPSCs from G2019S heterozygotes display the transferrin dysregulation when exposed to inflammatory stress, consistent with the phenotype observed in LPS-treated G2019S KI mice. Technically the approaches are sound and the results are robust. The significance of these studies is high given that the field has maintained that LRRK2 function in brain microglia is limited and unimportant; yet these data underscore its role in stress responses, a role inherent in the fact that LRRK2 is a member of the RIP kinase family and these are regulators of cellular stress.

Thank you!
