# Supplementary material for: Mutations in LRRK2 linked to Parkinson disease sequester Rab8a to damaged lysosomes and regulate transferrin-mediated iron uptake in microglia
Source: PLoS Biol. 2021 Dec 16;19(12):e3001480. doi: 10.1371/journal.pbio.3001480 (PMC8675653; doi:10.1371/journal.pbio.3001480)
Supplement: Attachment — Submitted filename: Response to Reviewers 2nd Revision.docx [file pbio.3001480.s015.docx]

Reviewer #1:

Figure 1A. Where is Rab8 in the wild type and MLi-2 treated cells? Rab8 is broadly distributed as shown in papers from many other labs and these images are overcorrected such that most of the Rab8 signal is no longer detected. The images don't look right. Row 4, why is the cell with poor LAMP2 staining selected? Same for E and G--cells have much more Rab8a, and nocodazole should redistribute it, not make it disappear. That it does disappear is a strong indication that the images have been overcorrected. In 1E, why is the red cell with a lot of LRRK2 staining not examined? Why do they include the totally nonspecific 188574 Rab8 antibody in their analyses (Fig. S1)? Are the effects only seen in 293T cells upon massive overexpression of LRRK2?

We first would like to state that we used the Cell Signaling 6975 antibody for all our analyses, which had distinct staining that was removed by siRNA treatment (Figure S1A) had a single monospecific band by western also blocked by siRNA treatment (Figure S1B). We did not use the abcam ab188574 antibody that has multiple non-specific bands for any staining presented here; the point of doing this validation was of course to not use a “totally nonspecific antibody” that we note has substantial cytoplasmic staining. We had previously discussed the validation data in response to reviewer 2, but have now also stated this clearly in S1 Figure legend and in the first paragraph of the results so hopefully this will no longer cause confusion.

Regarding the correction of images, in HEK293T cells, endogenous Rab8a staining is distinct and bright at structures resembling tubular endosomes and lysosomes, while being very faint in the cytoplasm. Thus, if we were to adjust the brightness to show cytoplasmic staining, Rab8a at the lysosome would appear saturated, losing detail of membrane localization. In compiling the confocal images we adjusted the brightness of Rab8a staining equally across all panels to ensure that Rab8a-positive lysosomes are not saturated and Rab8a staining at the recycling endosomes/lysosomal membrane is clearly visible (as also seen by super-resolution microscopy in Figure 1D).

We are not trying to imply that Rab8a is not in other compartments, However, our staining is in line with early publications from the Caplan group who reported association of Rab8a with tubular endosomes in HeLa cells:.

Mahak Sharma, Sai Srinivas Panapakkam Giridharan, Juliati Rahajeng, Naava Naslavsky, Steve Caplan. MICAL-L1 links EHD1 to tubular recycling endosomes and regulates receptor recycling. Mol Biol Cell. 2009 Dec;20(24):5181-94.

Joseph T Roland , Anne K Kenworthy, Johan Peranen, Steve Caplan, James R Goldenring. Myosin Vb interacts with Rab8a on a tubular network containing EHD1 and EHD3. Mol Biol Cell 2007 Aug;18(8):2828-37.

Finally, to demonstrate how our images have not been over-corrected in export, we attach below an example of how Rab8a staining looks upon increasing the brightness, and comparing Rab8a siRNA to NTC siRNA treated HEK293 cells. What is evident is that when exporting the image with increased brightness we begin to see background staining that is present in both control and Rab8a siRNA treated cells. This gives us the confidence that the exported images that we include in figure 1 demonstrate real Rab8a staining.

In terms of whether “massive over-expression” is required to sequester Rab8a onto lysosomes, other groups have shown that endogenous Rab8a can localize onto lysosomes without exogenous LRRK2 expression, at least under conditions of lysosomal damage (Eguchi et al, 2018; PNAS). Here, we showed that pathogenic LRRK2 mutants have the ability to drive Rab8a to lysosomes to a much greater extent than wild type protein, although we predict that if there was enough activity and expression then wild type LRRK2 would be able to support such events. Hence, exogenous expression was necessary to explore the mutations as well to facilitate staining for LRRK2 in HEK293T cells that lack endogenous LRRK2. That we are able to show mutant specific effects argues that these effects are not simply driven by overexpression.

Nevertheless, to validate our results in a different cell model, per comments on critical experiments from reviewer 2, we have now performed Rab8a localization analysis in primary mouse astrocytes where overexpression levels are modest. In this experiment primary astrocytes were transfected with HaloTag pDEST G2019S LRRK2, visualized by addition of JFX650 ligand, along with GFP-Rab8a and RFP-Lamp1 (Fig S3A). We compared live imaging to standard immunocytochemistry and in both experimental paradigms and have detected Rab8a at lysosomes in the presence of G2019S LRRK2 (Fig S3). Of note, in astrocytes, we do not see much evidence of localization to pericentrin-positive structures, suggesting some cell-type contribution to relative lysosomal recruitment of Rab8a and relevant to the next point.

Fig. S4. The purpose of nocodazole is to help demonstrate co-localization--that two markers move together because they are in the same compartment. The authors' own data show loss of colocalization upon nocodazole, which demonstrates that the markers are in DIFFERENT compartments, not that microtubule based motors are needed to move things.

We respectfully disagree that a partial decrease in colocalization indicates invalidation of our overall conclusion that some Rab8a is present at lysosomes. In the prior round of review it was suggested that there might be some adventitious staining of Lamp1 positive structures because Rab8a is at centrioles that have spatially proximate lysosomes and that we should identify the fraction of Rab8a that colocalizes with a lysosomal marker -/+ nocodazole. We present data staining for pericentrin (now fig S3A,B) and Lamp2 (S3C, D) in HEK cells. Nocodazole treatment does induce change in Rab8a/LAMP2 co-staining as evaluated by Mander’s coefficient but there is still a substantial overlap between Rab8a and LAMP2. We have now added an extra representative image of Rab8a staining that shows clear association with Lamp2 under nocodazole treatment, in HEK293T cells (Fig S3C lower panel). One aspect to note is that nocodazole itself changes LRRK2 localization, something we have in fact reported many years ago (Greggio et al., 2006) in HEK cells overexpressing LRRK2 where strong punctate structures are found to disperse after microtubule depolymerization. Given the intervening work from Hilfiker’s lab and others and our own staining with this phenomenon can also be seen in figure S3 in the current manuscript. We therefore suggest that the retained partial colocalization of Rab8a and Lamp2 when microtubules are disrupted and centrosome separation increased supports that, in HEK cells, some LRRK2 is authentically at lysosomes.

However, we also acknowledge that the level of resolution of the light microscopy methods may be inadequate to fully resolve two nearby compartments. We therefore additionally performed super resolution microscopy using the validated antibodies in primary mouse astrocytes. We used a second cell type based on advice from reviewer 2 (Fig 1E and F). These results demonstrated that Rab8a can co-localize with lysosomes and only modestly with centrosomes in this non-transformed cell type. As outlined in our response to reviewer 2 below, this strengthens our observation that Rab8a is recruited to lysosomal membranes and suggests that certain Rab8a phenotypes can be cell-type specific. As we write in the revised text, it is reasonable to interpret our data as showing at least two pools of Rab8a, one centriolar and one lysosomal. In light of the new data in primary astrocytes that were suggested by reviewer 2, we have moved the Rab8a staining in nocodazole-treated cells panel to supplementary figure 3.

"The interaction of Rab8a with the GDI was blocked by T22N and enhanced by Q67L." GDI does not bind at all to GTP-bearing Rabs so this is the opposite of what a huge body of work tells us should have been seen. Indeed, the figure 3 shows the opposite; this reviewer is surprised they saw any binding to Q67L. As for MICAL-L1 binding, it should have strongly enhanced by Q67L mutation and isn't so not sure what to conclude from data--perhaps it is because the IPs were done in EDTA with no magnesium or nucleotide.

The overall tenor of the reviewer’s comments is that the revised manuscript was not responsive to prior review. However, the only comment relating to this experiment was ”Next the authors confirm that pRab8a cannot bind Rabin8 but binds MICAL-L1. This confirms a report by Steger., 2017 using mass spec IP, and is consistent with the colocalization in cells shown here ±LRRK2.” Hence, the prior concern was not that the presented data was against the literature but instead that part of it replicates known events. However, we are happy to agree with the reviewer that the other half of the data including the Q67L Rab8a mutant does not behave as expected in terms of binding to GDI or MICAL-L1. We have therefore now removed this panel from this work in the updated fig 3. Thus, overall, we have now responded to different concerns raised in different rounds of review from this reviewer.

Transferrin uptake -- the authors have not addressed the issues raised. If they want to conclude there are differences in uptake, they need to explain how and why. More surface receptors? They say no. More recycling of receptors to the surface? They should follow the release of Tf in the same experiment as the uptake to be sure there are no differences in cell number or confluency that could differ between lines and change appearance of different mutants since the differences are so small.

We would like to point out here that level of mechanism is rarely fully resolved and that there is validity to robustly characterized observations. Based on prior comments from all reviewers we have shown that Tf vesicles are significantly more clustered in cells expressing G2019S LRRK2 compared to WT LRRK2 and this is ameliorated by the potent LRRK2 inhibitor MLi-2 (supplementary figure S5D). Furthermore, we show that Tf:LAMP2 colocalization is diminished by Rab8a knockdown (FIgure 4J). Similarly, expression of kinase-hyperactive mutant LRRK2 induces clustering of TfR vesicles (figure 4A-C) and increase in TfR:Lamp1 colocalization compared to WT LRRK2 (Figure S5A, B). In the first round of reviews we also examined the effect of nocodazole on TfR colocalization with Lamp2 in cells expressing mutant LRRK2. While our data suggested that nocodazole may affect the association of TfR with lysosomal structures, we note that nocodazole affects both lysosomal positioning and LRRK2 cellular distribution. Based on this and the remarks of the reviewer on interpreting nocodazole treatment experiments we have now removed panel B (nocodazole treatment part) from supplementary figure 5 for this submission due to difficulty in interpretation..

Whether this set of observations represents sufficient mechanism is ultimately an editorial decision. However, we would point out that this is not dissimilar to the level of detail required for other high profile publications. As a random example, in a recent paper in eLife, Berndsen et al showed that the phosphatase PPM1H counteracts LRRK2 using a variety of knockout and overexpression approaches in one cell line (A549 cells). In that paper there was a very nice mutation used but several aspects of mechanism such as the relation between Golgi and cilia that were not addressed, even in the in vitro model. Thus, we feel that we have delineated a reasonable interpretation of our available observations but acknowledge that aspects of the model can be clarified and expanded in the future. Having said this, we have now specifically examined cell surface TfR levels by surface biotinylation/enrichment and western blot analysis in HEK293T cells stably expressing WT and G2019S LRRK2. We did not detect a difference in surface TfR levels or total TfR levels between the genotypes. We have added this panel in figure S5E. An effect of LRRK2 mutations on clathrin-mediated endocytosis has been documented in different cell models by multiple independent groups (refs below; please note that we did not include additional examples of synaptic vesicle endocytosis) and thus whether the results are sufficient to explain all aspects of LRRK2 effects on transferrin receptor, they would appear to be robust. Our current model is that LRRK2 affects how TfR is handled in endolysosomes and this is not reflected in surface receptor levels but rather in intracellular localization of the receptor in respect to lysosomes.

Liu Q, Bautista-Gomez J, Higgins DA, Yu J, Xiong Y. Dysregulation of the AP2M1 phosphorylation cycle by LRRK2 impairs endocytosis and leads to dopaminergic neurodegeneration. Sci Signal. 2021 Jul 27;14(693):eabg3555. doi: 10.1126/scisignal.abg3555. PMID: 34315807.

Heaton GR, Landeck N, Mamais A, Nalls MA, Nixon-Abell J, Kumaran R, Beilina A, Pellegrini L, Li Y; International Parkinson Disease Genomics Consortium (IPDGC), Harvey K, Cookson MR. Sequential screening nominates the Parkinson's disease associated kinase LRRK2 as a regulator of Clathrin-mediated endocytosis. Neurobiol Dis. 2020 Jul;141:104948. doi: 10.1016/j.nbd.2020.104948. Epub 2020 May 17. PMID: 32434048; PMCID: PMC7339134.

Connor-Robson N, Booth H, Martin JG, Gao B, Li K, Doig N, Vowles J, Browne C, Klinger L, Juhasz P, Klein C, Cowley SA, Bolam P, Hirst W, Wade-Martins R. An integrated transcriptomics and proteomics analysis reveals functional endocytic dysregulation caused by mutations in LRRK2. Neurobiol Dis. 2019 Jul;127:512-526.

Cha SH, Choi YR, Heo CH, Kang SJ, Joe EH, Jou I, Kim HM, Park SM. [Loss of parkin promotes lipid rafts-dependent **endocytosis** through accumulating caveolin-1: implications for Parkinson'**s** disease.](https://pubmed.ncbi.nlm.nih.gov/26627850/) Mol Neurodegener. 2015 Dec 1;10:63

Rivero-Ríos P, Gómez-Suaga P, Fernández B, Madero-Pérez J, Schwab AJ, Ebert AD, Hilfiker S. [Alterations in late endocytic trafficking related to the pathobiology of **LRRK2**-linked Parkinson'**s** disease.](https://pubmed.ncbi.nlm.nih.gov/26009181/) Biochem Soc Trans. 2015 Jun;43(3):390-5

Abstract - "Here, we show that gain-of-function mutations in LRRK2 induce sequestration of endogenous Rab8a into lysosomes in cells". The authors have not shown Rab8 sequestration as proven by lack of Rab8/LRRK2/LAMP colocalization in the presence of nocodazole, nor have they shown Rab8a to be "into" the lysosome. If Rab8a is in the lysosome, it has other implications.

We have corrected this poor wording in the abstract.

There is inconsistency in cell outlines between figures, some have and others don't have them. figures 1A cell outlines don't match the actual cell staining.

Figure legends - The figure legends need to clearly explain what the figures actually describe not the results. Terms such as "cytocenter" in Fig1 legend are not explained. Legends of most figures (as well as the main text) need to indicate the type of cells used for each experiment. The number of cells and vesicles counted have been noted, but not whether standard errors from multiple experiments have been plotted.

The legends have been updated to indicate the type of cells and reworded to clearly explain what the figures describe according to the reviewer’s recommendations.

Vesicle quantitation - Pictures in Fig 6 do not reflect the colocalization numbers quantified with Pearson's (40% colocalization). Distribution of the vesicles and their relative distance from each other are also a function of the size and spreading of the cell. A smaller cell would naturally have more "clustered" vesicles as is seen in Fig 6A +/- LPS. Are these distances normalized for cell size?

We thank the reviewer for this comment. We examined cell sizes throughout WT and G2019S LRRK2 microglia treated with DMSO or LPS and reported this in figure S6E. We observed an increase in cell size with LPS treatment and a similar spread of cell area between WT and G2019S LRRK2 cells, suggesting that any changes in vesicle distribution are not driven by differences in total cell size. This does not exclude cell shape and nucleus size as potential confounding factors.

Fig 3D/E - Have the authors looked at MICAL or MICAL-L1 in their western blots? These are two different proteins. The blots need to be quantified and replicates plotted to show (no) changes in interactions after immune precipitation. Some of the signals shown don't make any sense.

MICAL-L1 has been corrected in the figure. We apologize for using the wrong protein name.

In Fig 1E/G, nocodazole treatment did not change perinuclear Rab8 accumulation. In S4 nocodazole treatment also dispersed TfR, LAMP and LRRK2. These data indicate that perinuclear clustered Rab8a, TfR, LAMP and LRRK2 are all on different vesicles, possibly in proximity until treated with nocodazole. This could explain why there is no difference in transferrin recycling or uptake in most of the experiments. Peri-nuclear transferrin / transferrin receptor accumulation is well known irrespective of pathogenic mutant LRRK2 activity (Maxfield et al 1984 Cell).

"Mutant LRRK2 expressing cells sequestered Tf in enlarged vesicles that were labelled with LRRK2 and Rab8a (Fig 4D) " - Sequestration cannot be concluded since data indicating transferrin being held in the perinuclear LRRK2-compartment longer for mutant than WT is not shown.

This has now been corrected to “Mutant LRRK2 expressing cells exhibited association of Tf with enlarged vesicles that were labelled with LRRK2 and Rab8a” to represent more accurately our observations.

"ICP-MS analysis revealed significantly elevated iron levels in cells stably-expressing G2019S LRRK2 compared to WT LRRK2 (Fig 4K)". I2020T has the strongest effect on TfR according to authors, yet iron accumulation is unaltered. Moreover an MLi-2 or kinase inhibitor experiment was not performed. What would the molecular basis be for the data in 4K anyway?

While mis-trafficking of TfR that is observed in cells over-expressing mutant LRRK2 is a potential mechanism driving dysregulation of iron in G2019S cells, LRRK2 may be affecting other pathways responsible for cellular iron regulation. As outlined in the discussion, lysosomes are physiological iron stores and lysosomal acidification mediates iron reduction and subsequent release into the cytoplasm. Impaired lysosomal acidification has been reported to trigger iron deficiency *in vivo*. As LRRK2 mutations have been reported to alter the autophagic lysosomal pathway in carriers as well as to perturb lysosomal acidification in knock-in mouse models, it is plausible that LRRK2 mutations affect cellular iron availability by modulating lysosomal acidification. This remains to be explored.

Fig 7/8 showing increased iron accumulation (along with Tf and Fth) post LPS stimulation in striatal regions is potentially novel and interesting, more so because of the enhanced effect in G2019S and decreased effect in KO mice. The authors tried looking at striatal microglia and found no changes in Fth1 nor Iba1 (Fig 8E/D). Neither the data nor the images support this statement - "striatal microglia accumulate higher levels of ferritin-bound iron upon inflammation by LPS compared to WT LRRK2 mice". This increase could be in other cell types though. The levels of transferrin receptor and dmt1 are very low in various cell types in brain.

We co-stained brain slices with ferritin heavy chain and Iba1 and found that around 50% of ferritin-positive cells were also positive for Iba1 (based on nuclei/FTH/Iba1 count). Importantly, a lack of an increase in the proportion of FTH positive Iba1 does not mean that FTH is not increased, rather that the number of cells expressing this marker is constant. We certainly acknowledge that other cell types may also accumulate iron following LPS-induced inflammation and drive the observed upregulation of ferritin-bound iron in G2019S LRRK2 knock-in mice compared to WT mice. This has now been clarified in the text (end of results section). Panel D is a representative image showing co-positivity of Iba1 and ferritin and was not used to quantify the levels of ferritin in microglia cells.

Reviewer #2:

Original comments: The authors have addressed all my comments, and I deem the manuscript suitable for publication in PLoS Biology in its present form.

After reading reviewer 1's feedback: I´ve carefully looked over the comments from referee 1 to both the initial and revised version of the manuscript. I think the present work is important, as revealing a link between pathogenic LRRK2 and deregulated iron handling, with the TfR likely accumulating in an endolysosomal compartment. In my opinion, the data in iPSC-derived microglia as well as in the G2019S knockin mouse model are of particular interest.

The bulk of the mechanistic work has been performed in HEK293 cells overexpressing pathogenic LRRK2, and often employing high-content imaging. I think this is where most of the critiques from referee 1 are coming from. HEK cells have a very small cytoplasm, such that any colocalization analysis becomes limited. The authors conveniently used HEK cells stably overexpressing wildtype and various LRRK2 mutants. I feel that some of the comments from referee 1 could easily be addressed using another cell type transiently transfected with tagged LRRK2 constructs and suited for quantitative high-resolution confocal image analysis. Some key experiments could be repeated to corroborate the author´s conclusions, and this would not entail a lot of additional work.

We thank the reviewer for the opportunity to further enhance our study. Following the reviewer’s recommendation we have now tested and validated our observations in different cell models. To this end, we used super-resolution microscopy to examine the recruitment of Rab8a to lysosomal structures in mouse primary astrocytes transiently expressing 3xFLAG-G2019S LRRK2 and compared that to association with centrosomes (pericentrin staining). In mouse primary astrocytes the majority of LRRK2 and Rab8a positive puncta were Lamp1 positive while we did not detect Rab8a at the centrosome (pericentrin staining) (new fig 1E and F). This strengthens our observation that Rab8a is recruited to lysosomal membranes and suggests that certain Rab8a phenotypes can be cell-type specific or affected by the levels of LRRK2 transient transfection.

Apart from this, I think there are two main issues. One is related to nocodazole treatment (an experiment proposed by referee 1 in the first round of comments). I was not surprised to see the data presented, as phospho-Rab8 is not only localized to lysosomes, but also stably associates with centrosomes, and we have shown that this interaction is not disrupted by nocodazole. So there are likely at least two pools of phospho-Rab8, one bound to the centrosome, and the other to lysosome-related structures. Importantly though, most permeabilization conditions cause rapid extraction of prenylated Rab proteins from membranes, which makes interpretation of such experiments difficult. A better way to address the nocodazole comment would be to employ a live imaging approach.

We have now performed live imaging analysis of Rab8a localization in mouse primary astrocytes (new fig1E and F) and compared it to standard immunocytochemistry. We expressed HaloTag pDEST G2019S LRRK2 and vizualised using the JFX650 ligand, along with GFP Rab8a and RFP-Lamp1. In our hands both fixed and live imaging showed clear association of Rab8a with lysosomes validating our main observation. We attempted to visualize Rab8a and centrosomes by live imaging using the dsRed-cent2 construct by Addgene (#29523) but the latter look cytoplasmic and did not give the expected centrosomal distribution under transient expression in mouse primary astrocytes. Thus, to answer this concern, we now show that a) Rab8a is recruited to lysosomes in primary astrocytes as well as Heks, b) Rab8a staining is indistinguishable in live imaging versus fixed/permeabilized condition showing clear lysosomal association, and c) Rab8a did not associate with pericentrin in astrocytes expressing G2019S LRRK2 (new fig1F). We believe that these additions support the view of the reviewer that two distinct pools of Rab8a exist in mutant LRRK2 expressing cells, at centrosomes and lysosomal-related structures, and this has been reflected in the text.

.

The second main issue is related to the reported perinuclear TfR clustering, and how this may relate to TfR trafficking alterations. Some of my comments to the initial version of the manuscript were related to this as well. In the revised version of the manuscript, the authors try to address this issue by performing uptake/recycling assays, and the data presented indicate very subtle alterations. One could argue that the effects observed do not warrant a link between altered TfR trafficking and pathogenic LRRK2. Several years ago, we looked at TfR trafficking in pathogenic LRRK2 expressing cells and only found subtle alterations. I feel that the data presented in this manuscript properly reflect what is going on, namely a subtle effect on TfR trafficking which is hard to dissect (uptake versus recycling versus surface levels of receptors). The question is whether this is enough to give rise to the altered iron handling as observed in vivo. I feel that the authors shy away from making strong statements related to this mechanistic link, even though a potential link is obviously inferred.

We agree with the reviewer that the effects of LRRK2 on transferrin signaling are subtle and may not fully explain the iron upregulation phenotype we see *in vivo*. This has been outlined in the discussion along with further experiments that may be helpful to delineate iron dyshomeostasis in the context of LRRK2 mutations.

Other comments from referee 1 could be addressed by rephrasing the manuscript, for example:

-mutations in Rab8a and differential binding to GDI or MICAL-L1: in some cases, these mutations do not properly mimic the GTP-bound versus GDP-bound version of a Rab protein. The data presented relating to MICAL-L1 have limited meaning, are not relevant for the conclusions of the manuscript, and could be removed altogether

Based on reviewer 1 and 2 comments on figure 3, we have now removed the co-IP experiments utilizing Rab8a mutants such as Q67L Rab8a. Since MICAL-L1 is an established interacting partner of Rab8a we left these data in the manuscript.

-abstract mentions "into" the lysosome, whilst they show recruitment of both proteins (LRRK2 and Rab8a) to the membrane of LAMP1-positive lysosomes. Careful rephrasing would solve this issue. Similarly, "cytocenter" is a flashy terminology, but not properly defined. Figure legends could be improved in terms of annotation of cells and vesicles counted, specifics related to standard errors which have been plotted, etc.

We have now corrected these instances, thank you.
