# Supplementary material for: Mutations in LRRK2 linked to Parkinson disease sequester Rab8a to damaged lysosomes and regulate transferrin-mediated iron uptake in microglia
Source: PLoS Biol. 2021 Dec 16;19(12):e3001480. doi: 10.1371/journal.pbio.3001480 (PMC8675653; doi:10.1371/journal.pbio.3001480)
Supplement: Attachment — Submitted filename: Response to reviewers 3.docx [file pbio.3001480.s016.docx]

Reviewer remarks:

Reviewer #2: I am satisfied with the author´s rebuttal and revised version of the manuscript, and have only minor comments which need to be addressed as indicated below:

1. Figure 1 E: the colocalization of Rab8a with LRRK2-G2019S in the live cell image is convincing, but not the colocalization of these two markers with LAMP1. Please replace with another image where the colocalization of all three markers is more evident.

We thank the reviewer for the opportunity to further enhance our study. We have now replaced this panel with a representative image where the colocalization is more evident.

2. Figure 4: Panel J is confusing. The authors state that "Knockdown of Rab8a expression rescued the increase in the proportion of Tf that co-localized with LRRK2 relative to total internalized Tf caused by mutant LRRK2". Whilst there is a clear a difference in the numbers from ctrl versus Rab8a RNAi in I2020T expressing cells, it would be good to include data from wt expressing cells. Otherwise, rephrase to state that knockdown of Rab8a decreased the proportion of Tf that co-localized with LRRK2, rather than a statement regarding "rescue".

We have corrected this in the text.

3. Results: "Lastly, ICP-MS analysis revealed elevated iron levels in cells stably-expressing G2019S LRRK2 compared to WT LRRK2". Please also state that the pathogenic R1441C and Y1699C mutants do not show detectable alterations in iron levels (Fig. 4K), at least as measured by ICP-MS.

This has now been amended.

4. The authors show increased colocalization of TfR with LAMP2 in pathogenic LRRK2-expressing cells (Figure S5A,B), and they show that LAMP2-positive structures are cathepsin D-negative (Figure 2A). Whilst their data support the notion that pathogenic LRRK2 causes TfR accumulation in damaged (degradation-deficient) lysosomes, it remains possible that the identity of the vesicular compartment where TfR accumulates is not lysosomal, but endosomal in nature (see eg. Cheng et al., JCB, 2018; 40% of LAMP1 can colocalize with the early endosomal marker EEA1 in the soma of cultured neurons). Perhaps a small mention in the discussion regarding this possibility is warranted.

We thank the reviewer for this suggestion, we have added this in the discussion.

5. Figure legend 1: (A)... stained for FLAG LRRK2, endogenous Rab8a and endogenous Lamp1. This should read Lamp2, not Lamp1.

We have now corrected this.

6. Methods, section immunocytochemistry: please specify antibodies used against LAMP1, LAMP2 and cathepsin D.

We have now specified these antibodies, thank you.

Data and other policy-related requests:

1) Title: We suggest the following title, which we think might be more appealing to a broad readership: "Mutations in LRRK2 linked to Parkinson’s disease sequester Rab8a to damaged lysosomes and regulate transferrin-mediated iron uptake in microglia." However, we would be happy to work with you on an alternative, if our recommendation misrepresents your findings.

Thank you for the suggestion, we are happy to use your chosen title. We have now updated the tile in the manuscript.

2) Blurb: Please provide a blurb which (if accepted) will be included in our weekly and monthly Electronic Table of Contents, sent out to readers of PLOS Biology, and may be used to promote your article in social media. The blurb should be about 30-40 words long and is subject to editorial changes. It should, without exaggeration, entice people to read your manuscript. It should not be redundant with the title and should not contain acronyms or abbreviations. For examples, view our author guidelines: <https://urldefense.proofpoint.com/v2/url?u=https-3A__journals.plos.org_plosbiology_s_revising-2Dyour-2Dmanuscript-23loc-2Dblurb&d=DwIGaQ&c=sJ6xIWYx-zLMB3EPkvcnVg&r=WxfJHriSH3E2KLDq8rhQh42Du1ps0PhYv-swM0HpfNRlhRLgPavYTHq1DNLDzr-9&m=BPmQoBytia4d_zfwTDsWrbgx42YFjBOCN3dgmB9kHJ0&s=v2HwR8ARuwAf_i4shcIWIzz1tp9L-H3fIsO0UxcnmO0&e=>

While brain iron deposition is a feature of Parkinson’s disease pathology, how this contributes to neurodegeneration is unknown. Here, Mamais and colleauges show aberrant brain iron accumulation in vivo and iron dyshomeostasis *in vitro* by LRRK2 mutations.

3) Data not shown: Please note that per journal policy, we do not allow the mention of "data not shown", "personal communication", "manuscript in preparation" or other references to data that is not publicly available or contained within this manuscript. Please either remove mention of these data or provide figures presenting the results and the data underlying the figure(s).

We have removed mention of the said data.

4) Blot and gel reporting requirements: We require the original, uncropped and minimally adjusted images supporting all blot and gel results reported in an article's figures or Supporting Information files. We will require these files before a manuscript can be accepted so please prepare and upload them now. Please carefully read our guidelines for how to prepare and upload this data: <https://urldefense.proofpoint.com/v2/url?u=https-3A__journals.plos.org_plosbiology_s_figures-23loc-2Dblot-2Dand-2Dgel-2Dreporting-2Drequirements&d=DwIGaQ&c=sJ6xIWYx-zLMB3EPkvcnVg&r=WxfJHriSH3E2KLDq8rhQh42Du1ps0PhYv-swM0HpfNRlhRLgPavYTHq1DNLDzr-9&m=BPmQoBytia4d_zfwTDsWrbgx42YFjBOCN3dgmB9kHJ0&s=zdDLlkdu_PfyBjI2qcREFbaI29I-VhhOdKABG-c6IVU&e=>

We have included all uncropped blots as supplementary figure S8.

5) Ethics:

5.a) Please include within your manuscript the ID number of your protocols approved by the Institutional Animal Care and Use Committee of National Institute on Aging, NIH.

The protocol ID has been added to the methods section.

5.b) Please include the specific national or international regulations/guidelines to which your animal care and use protocol adhered. Please note that institutional or accreditation organization guidelines (such as AAALAC) do not meet this requirement.

We have now added this information in the “Animal procedures” section of our methods.

6) Data: You may be aware of the PLOS Data Policy, which requires that all data be made available without restriction: <https://urldefense.proofpoint.com/v2/url?u=http-3A__journals.plos.org_plosbiology_s_data-2Davailability&d=DwIGaQ&c=sJ6xIWYx-zLMB3EPkvcnVg&r=WxfJHriSH3E2KLDq8rhQh42Du1ps0PhYv-swM0HpfNRlhRLgPavYTHq1DNLDzr-9&m=BPmQoBytia4d_zfwTDsWrbgx42YFjBOCN3dgmB9kHJ0&s=LSpxNWTpSX6uL1A93AumyXFvHJ0rM8-SBNEdq6wMiz4&e=> . For more information, please also see this editorial: <https://urldefense.proofpoint.com/v2/url?u=http-3A__dx.doi.org_10.1371_journal.pbio.1001797&d=DwIGaQ&c=sJ6xIWYx-zLMB3EPkvcnVg&r=WxfJHriSH3E2KLDq8rhQh42Du1ps0PhYv-swM0HpfNRlhRLgPavYTHq1DNLDzr-9&m=BPmQoBytia4d_zfwTDsWrbgx42YFjBOCN3dgmB9kHJ0&s=6JBZthSpsXJUTUHt4tHQV89zq4EJL1m96Sl_PIqayWE&e=>

Note that we do not require all raw data. Rather, we ask for all individual quantitative observations that underlie the data summarized in the figures and results of your paper. For an example see here: <https://urldefense.proofpoint.com/v2/url?u=http-3A__www.plosbiology.org_article_info-253Adoi-252F10.1371-252Fjournal.pbio.1001908-23s5&d=DwIGaQ&c=sJ6xIWYx-zLMB3EPkvcnVg&r=WxfJHriSH3E2KLDq8rhQh42Du1ps0PhYv-swM0HpfNRlhRLgPavYTHq1DNLDzr-9&m=BPmQoBytia4d_zfwTDsWrbgx42YFjBOCN3dgmB9kHJ0&s=gFr-x1E6VVFoEs5M5HXpMjhMsrVcAFMbVDGgEedPtKE&e=>

These data can be made available in one of the following forms:

I) Supplementary files (e.g., excel). Please ensure that all data files are uploaded as 'Supporting Information' and are invariably referred to (in the manuscript, figure legends, and the Description field when uploading your files) using the following format verbatim: S1 Data, S2 Data, etc. Multiple panels of a single or even several figures can be included as multiple sheets in one excel file that is saved using exactly the following convention: S1_Data.xlsx (using an underscore).

II) Deposition in a publicly available repository. Please also provide the accession code or a reviewer link so that we may view your data before publication.

Regardless of the method selected, please ensure that you provide the individual numerical values that underlie the summary data displayed in the following figure panels: Figures 1BCF, 2B, 3B, 4BDEFGJK, 5CDFGHI, 6BCDEF, 7C, 8BC, S2BC, S3BD, S4BC, S5BDE, S6BCE, and S7BD.

We have included all data as multiple sheets of one excel file: S1_Data.xlsx. The data presented in figure 5 were uploaded onto GEO and referenced in the manuscript.

NOTE: the numerical data provided should include all replicates AND the way in which the plotted mean and errors were derived (it should not present only the mean/average values).

6.a) Please also ensure that each figure legend in your manuscript includes information on where the underlying data can be found and that your supplemental data file/s has/have a legend.

6.b) Please ensure that your Data Statement in the submission system accurately describes where your data can be found.

------------------------------------------------------------------------
