# Supplementary material for: Mutations in LRRK2 linked to Parkinson disease sequester Rab8a to damaged lysosomes and regulate transferrin-mediated iron uptake in microglia
Source: PLoS Biol. 2021 Dec 16;19(12):e3001480. doi: 10.1371/journal.pbio.3001480 (PMC8675653; doi:10.1371/journal.pbio.3001480)
Supplement: Attachment — Submitted filename: Response to reviewers 4.docx [file pbio.3001480.s017.docx]

Remarks:

1) The academic editor is now satisfied with the quality of the images that you have provided as well as with the other revisions. However, s/he notes that you should i) indicate the total number of experiments for each figures, and ii) more clearly state in all figure legends whether the “n” represents true replicates (independent experiments) or pseudo replicates (cells or images from a single/few experiments). For example, we understand that in figures 1B and C, “n” does not represent independent experiments but cells that could be obtained from one or few experiments.

We have now explicitly stated the N and nature of replicates for each experiment presented.

2) Please also ensure that each figure legend in your manuscript includes information on where the underlying data can be found. You can write, for example, “The underlying data can be found in S1 Data/GEO accession numbers GSE186483 and GSE186559”

Done.

3) Please incorporate your “data not shown” into a supporting figure, updating the data files as well. There is at least one case of “data not shown”: “Mature iPSC-derived microglia were characterized by western blot and qPCR for expression of Iba1 protein and AIF1, TMEM119 and P2RY12 mRNA, respectively. (data not shown).”

As qRT-PCR characterization data of iMicroglia for expression of microglial markers has been included in a separate publication (under review), we excluded mention of these data here. We did however include characterization of Iba1 expression by WB and ICC (Figure S6). The text has been amended to reflect this and “(data not shown)” has now been removed.

4) Please update Figure S8 to include the raw image of the gels shown in Figure S1B.

Done.
